# Moderating effect of education on glymphatic function and cognitive performance in mild cognitive impairment

**DOI:** 10.3389/fnagi.2024.1399943

**Published:** 2024-05-02

**Authors:** Liang Zhou, Wenxia Yang, Yang Liu, Yu Zheng, Xin Ge, Kai Ai, Guangyao Liu, Jing Zhang

**Affiliations:** ^1^Department of Magnetic Resonance, The Second Hospital of Lanzhou University, Lanzhou, China; ^2^Second Clinical Medical School, Lanzhou University, Lanzhou, China; ^3^Philips Healthcare, Xi’an, China; ^4^Gansu Province Clinical Research Center for Functional and Molecular Imaging, Lanzhou, China

**Keywords:** cognitive reserve, glymphatic function, cognitive function, mild cognitive impairment, diffusion tensor image

## Abstract

**Objective:**

This research aims to investigate putative mechanisms between glymphatic activity and cognition in mild cognitive impairment (MCI) and analyzes whether the relationship between cognitive reserve (CR) and cognition was mediated by glymphatic activity.

**Methods:**

54 MCI patients and 31 NCs were enrolled to evaluate the bilateral diffusivity along the perivascular spaces and to acquire an index for diffusivity along the perivascular space (ALPS-index) on diffusion tensor imaging (DTI). The year of education was used as a proxy for CR. The ALPS-index was compared between two groups and correlation analyses among the ALPS-index, cognitive function, and CR were conducted. Mediation analyses were applied to investigate the correlations among CR, glymphatic activity and cognition.

**Results:**

MCI group had a significantly lower right ALPS-index and whole brain ALPS-index, but higher bilateral diffusivity along the y-axis in projection fiber area (Dyproj) than NCs. In MCI group, the left Dyproj was negatively related to cognitive test scores and CR, the whole brain ALPS-index was positively correlated with cognitive test scores and CR. Mediation analysis demonstrated that glymphatic activity partially mediated the correlations between CR and cognitive function.

**Conclusion:**

MCI exhibited decreased glymphatic activity compared to NCs. CR has a protective effect against cognitive decline in MCI, and this effect may be partially mediated by changes in glymphatic activity.

## Introduction

The glymphatic system is a highly polarized convective system of cerebrospinal fluid (CSF) and interstitial fluid exchange along the periarterial space (Nedergaard and Goldman, 2020). It is similar to the lymphatic system of the surrounding tissue and plays an important role in protein clearance and reduction of abnormal aggregation, such as the amyloid-β protein (Aβ) and Tau proteins. As mentioned in the literature review, the impairment of glymphatic system is a part of the pathophysiological mechanisms that mediate and accelerate the progression of neurodegenerative diseases, which is closely correlated to the cognition, executive function and memory in Alzheimer’s disease (AD) and AD-related dementia (Bah et al., 2023). In recent years, researchers have gradually used the non-invasive diffusion tensor imaging along the perivascular space (DTI-ALPS) to evaluate the function of glymphatic system (van der Thiel et al., 2023). DTI-ALPS can calculate the difference of diffusion rate of water molecules in different directions of diffusion in brain without contrast enhancement (Taoka et al., 2017). Although the ALPS-index reveals the diffusivity of perivascular spatial orientation in the periventricular white matter (WM), it is considered an indirect measure of the state of glymphatic function (Carlstrom et al., 2022). Studies have shown a positive relation between the ALPS-index and Mini-Mental State Examination (MMSE) scores, suggesting that the completeness of glymphatic function may play a role in cognitive prediction. Several reports involving patients with AD and mild cognitive impairment (MCI) have shown that the ALPS-index was related to Aβ protein in the CSF, fluorodeoxyglucose metabolism and cognitive function (Kamagata et al., 2022; Hsu et al., 2023; Zhang et al., 2023). The results of current studies on DTI-ALPS in MCI are inconsistent, with most studies finding a reduced ALPS-index in MCI compared to normal controls (NCs) (Steward et al., 2021; Liang et al., 2023), but some research have shown that there is no difference on ALPS-index between MCI and NCs after adjusting for education level (Kamagata et al., 2022).

Cognitive reserve (CR) is a proposed concept to elucidate the discrepancy between pathological changes and functional alterations in the brain. It is believed to act as a potential protective factor for preventing AD, enabling some individuals to maintain cognitive abilities and decelerate the progression of dementia (Stern and Barulli, 2019). Studies have demonstrated that individuals with MCI who possess higher CR levels are more likely to revert to normal rather than progress to dementia (Iraniparast et al., 2022) and have a beneficial impact on ameliorating cognitive impairment (Berezuk et al., 2021; Corbo et al., 2023). The level of education is commonly utilized as a proxy for CR in current researches and exhibits a strong correlation to dementia (Livingston et al., 2020). Enhancing educational attainment has been linked to a decreased risk of MCI and a postponement in the onset of clinical symptoms and disease progression (Liu et al., 2013). Neuroimaging researches have indicated that CR is linked to enhanced connectivity within cognitive control networks, particularly between the left frontal cortex and the dorsal attentional network in MCI (Franzmeier et al., 2017a,b). Additionally, it has been demonstrated that CR could modify cortical architecture and WM macromolecular volume, enhance cerebral blood flow, which may alleviate cognitive decline in MCI (Fingerhut et al., 2022; Serra et al., 2022; Brenner et al., 2023; Zhou et al., 2024).

To date, very little attention has been paid to the role of CR on glymphatic activity, and the relationship between glymphatic activity and CR in MCI is unclear. Therefore, in this study, we aimed to investigate glymphatic activity in MCI by the DTI-ALPS method and to explore the association between CR and glymphatic activity.

## Materials and methods

### Participants

Eighty-five subjects were included from the memory disorder clinic at the Department of Neurology in Lanzhou University Second Hospital and the local community, including 54 individuals with MCI and 31 NCs. The MCI subjects were classified according to the diagnostic criteria (Albert et al., 2011), and the Clinical Dementia Rating Scale (CDR) score was less than 0.5. Subjects met criteria for NCs based on: (1) over 50 years of age; (2) normal physical health; (3) have normal cognition, with a minimum score of 27–30 points on the MMSE; (4) CDR score of 0 point; (5) have no history of memory decline; and (6) right-handed. This research has received approval from the Ethics Committee of Lanzhou University Second Hospital, and all participants have provided written informed consent.

### Cognitive and cognitive reserve assessment

We used the Montreal Cognitive Assessment (MoCA) and the MMSE to assess general cognition. Memory recall was assessed using the Auditory Verbal Learning Test (AVLT, Chinese version), while verbal fluency was measured using the verbal fluency test (VFT). The activity of daily living (ADL) and instrumental activity of daily living (IADL) were used to assess subject’s ability in daily life. The years of education attained by an individual, as well as any vocational training completed, were used as proxy for CR, and training courses lasting at least 6 months were accounted for as 0.5 points.

### MRI imaging

All participants underwent brain scanning using an MRI-3 T machine (Ingenia CX, Philips Healthcare, Netherlands) equipped with a 32-channel head coil. The parameters for T1-weighted images of the whole brain were as followed: TR = 5.9 ms, TE = 3.7 ms, flip angle = 8°, FOV = 256 × 256 mm^2^, and voxel size = 1 × 1 × 1 mm. The parameters of the DTI sequence were as follows: TR = 5,000 ms, TE = 102 ms, spatial resolution = 2 × 2 × 2 mm^3^, b-value =1,000 s/mm^2^ along 120 gradient directions. A high-resolution 3D-T2 weighted image was also conducted to rule out brain disorders such as strokes or tumors with parameters: TR = 3,000 ms, TE = 250 ms, FOV = 256 × 256 mm^2^, and voxel size = 1 × 1 × 1 mm. Total intracranial volume was processed from the whole-brain T1-weighted images segmentation using the Computational Anatomy Toolbox 12(CAT-12), a toolbox of Statistical Parametric Mapping version 12 software package (SPM-12, https://www.fil.ion.ucl.ac.uk/spm/software/spm12/). The ALPS-index was calculated from DTI by FMRIB’s Software Library (FSL) 6.0.3 software package (Smith et al., 2004) and to estimate the glymphatic activity in each subject in accordance with previous studies (Taoka et al., 2017; Yokota et al., 2019). Diffusivity maps along the x, y-axis in projection fiber area and the diffusivity along the z-axis in association fiber area were performed by the color-coded 1st eigenvector maps on FSLEyes.^1^ At the level of the lateral ventricle body, two 5-mm-diameter spherical regions of interests (ROIs) were placed in the area of the projection fibers and association fibers on Mango software.^2^ The ALPS-index is defined by the ratio of mean of the diffusivity along the x-axis in projection fiber area (Dxproj) and diffusivity along the x-axis in association fibers area (Dxassoc) to the mean of diffusivity along the y-axis in projection fiber area (Dyproj) and diffusivity along the z-axis in association fibers area (Dzaccoc) as following formula: ALPS-index = mean (Dxproj, Dxassoc)/mean (Dyproj, Dzassoc). A larger ALPS-index indicates a larger rate of water diffusion along the perivascular space, while a value close to 1 suggests that the effect of water diffusion along the perivascular space is minimized.

### Statistical analyses

Group comparisons of clinical and cognitive function, and diffusivities, as well as correlation analysis between differential diffusivity and ALPS-index with CR, were performed using SPSS 22 software. We used the independent sample t-test for normal distribution data, while the Mann–Whitney U test was used for non-normal distribution data. Pearson’s correlation and Spearman correlation values were calculated to assess the correlation among the glymphatic activity, CR, and cognitive function. Mediation analysis was conducted using a SPSS plugin called “Process” (version 4.1), with age considered as an exposure variable, education as a predictor, glymphatic activity as a mediator, and cognitive test scores as outcomes. A *p*-value of less than 0.05 represents a significant difference.

## Results

### Demographic data and cognitive function

As presented in Table 1, age, sex, ADL, IADL, and geriatric depression scale (GDS) did not differ between MCI and NCs. The MCI group showed lower cognitive performance and total intracranial volume (TIV) than NCs group (*p* < 0.001), and had a significantly lower education level on average than the NCs group.

**Table 1:** Demographic characteristics.

	NCs (*n* = 31)	MCI (*n* = 54)	*p*
Age (y)	61.61 (5.8)	64 (60–68)	0.103
Gender (m/f)	9/22	17/37	0.814
Education (y)	11.32 (2.4)	8.5 (5.75–10.25)	<0.001*
MoCA	26 (26–27)	21 (17.75–23)	<0.001*
MMSE	28 (27–29)	25 (23–26)	<0.001*
AVLT-delay	9 (9–11)	4 (3–5)	<0.001*
VFT	15 (18–20)	13.5 (11.75–15)	<0.001*
ADL	8 (8–8)	8 (8–8)	0.601
IADL	12 (12–12)	12 (12–13)	0.11
GDS	2 (1–6)	1 (1–5.25)	0.481
TIV (cm^3^)	1366.26 (113.89)	1353.21 (1279.95–1501.24)	0.024*

Normal distribution data are presented as mean (SD); non-normal distribution data are presented as median (interquartile range); NCs, normal controls; MCI, mild cognitive impairment; MMSE, mini-mental state examination; MoCA, Montreal cognitive assessment; AVLT, auditory verbal learning test; VFT, verbal fluency test; ADL, activity of daily living; IADL, instrumental activity of daily living; GDS, geriatric depression scale; TIV, total intracranial volume, * *p*<0.05.

### Comparison of the diffusivities between two groups

**Figure 1:**
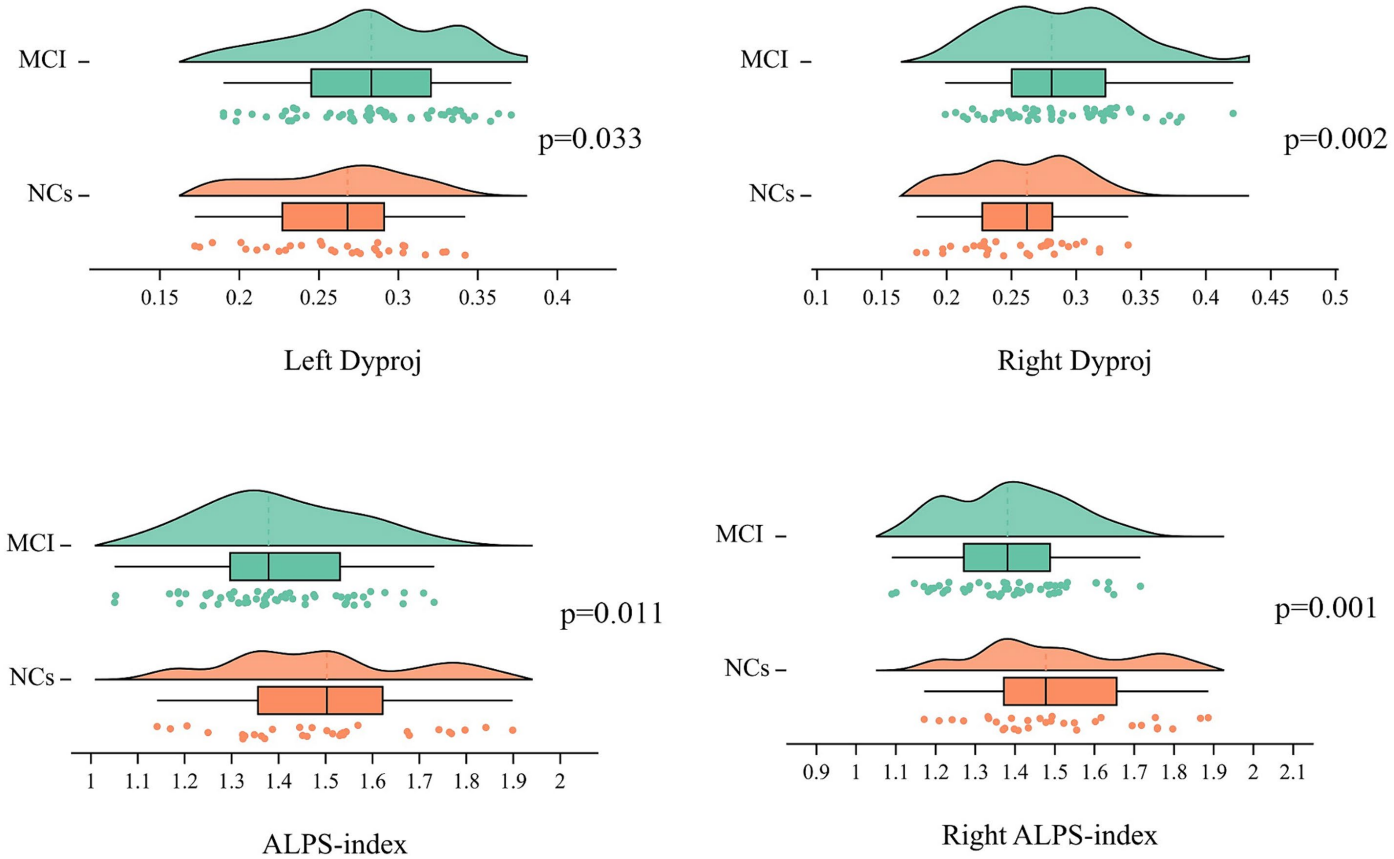
Distribution of the ALPS-index and bilateral Dyproj in MCI and HCs group. ALPS, diffusion tensor imaging along the perivascular space; Dyproj, diffusivity along the y-axis in projection fiber area; diffusivity was presented as apparent diffusion coefficient values (× 10^−3^ mm^2^/s). P, *p*-value.

Table 2 showed the comparison of the diffusivities between MCI groups and NCs, adjusting for age, gender and TIV, the MCI group had significantly lower right ALPS-index and whole brain right ALPS-index, but higher bilateral diffusivity along the y-axis in projection fiber area (Dyproj) than the NCs group (Figure 1). There was no significant difference in bilateral Dxassoc, Dzassoc, and Dxproj between the two groups.

**Table 2:** Comparison of the diffusivities between MCI patients and NCs.

Diffusivity	NCs	MCI	*p*
Left Dxproj	0.29 (0.03)	0.3 (0.27–0.32)	0.055
Left Dyproj	0.26 (0.05)	0.28 (0.05)	0.033*
Left Dxassoc	0.41 (0.04)	0.4 (0.04)	0.677
Left Dzassoc	0.21 (0.05)	0.21 (0.19–0.26)	0.322
Right Dyproj	0.25 (0.04)	0.29 (0.05)	0.002*
Right Dxproj	0.3 (0.27–0.3)	0.29 (0.27–0.33)	0.503
Right Dxassoc	0.39(0.05)	0.39 (0.04)	0.787
Right Dzassoc	0.2 (0.04)	0.21 (0.19–0.24)	0.274
Left ALPS-index	1.49 (0.22)	1.38 (1.29–1.58)	0.097
Right ALPS-index	1.51 (0.2)	1.38 (0.15)	0.001*
ALPS-index	1.5 (0.2)	1.4 (0.16)	0.011*

Normal distribution data are presented as mean (SD); non-normal distribution data are presented as median (interquartile range); NCs, normal controls; MCI, mild cognitive impairment; Dxassoc/Dzassoc, diffusivity along the x-axis/z-axis in association fiber area; Dyproj/Dxproj, diffusivity along the y-axis/x-axis in projection fiber area; diffusivity was presented as apparent diffusion coefficient values (× 10^−3^ mm^2^/s); * *p*<0.05.

### Correlation between the DTI-ALPS and cognition

**Figure 2:**
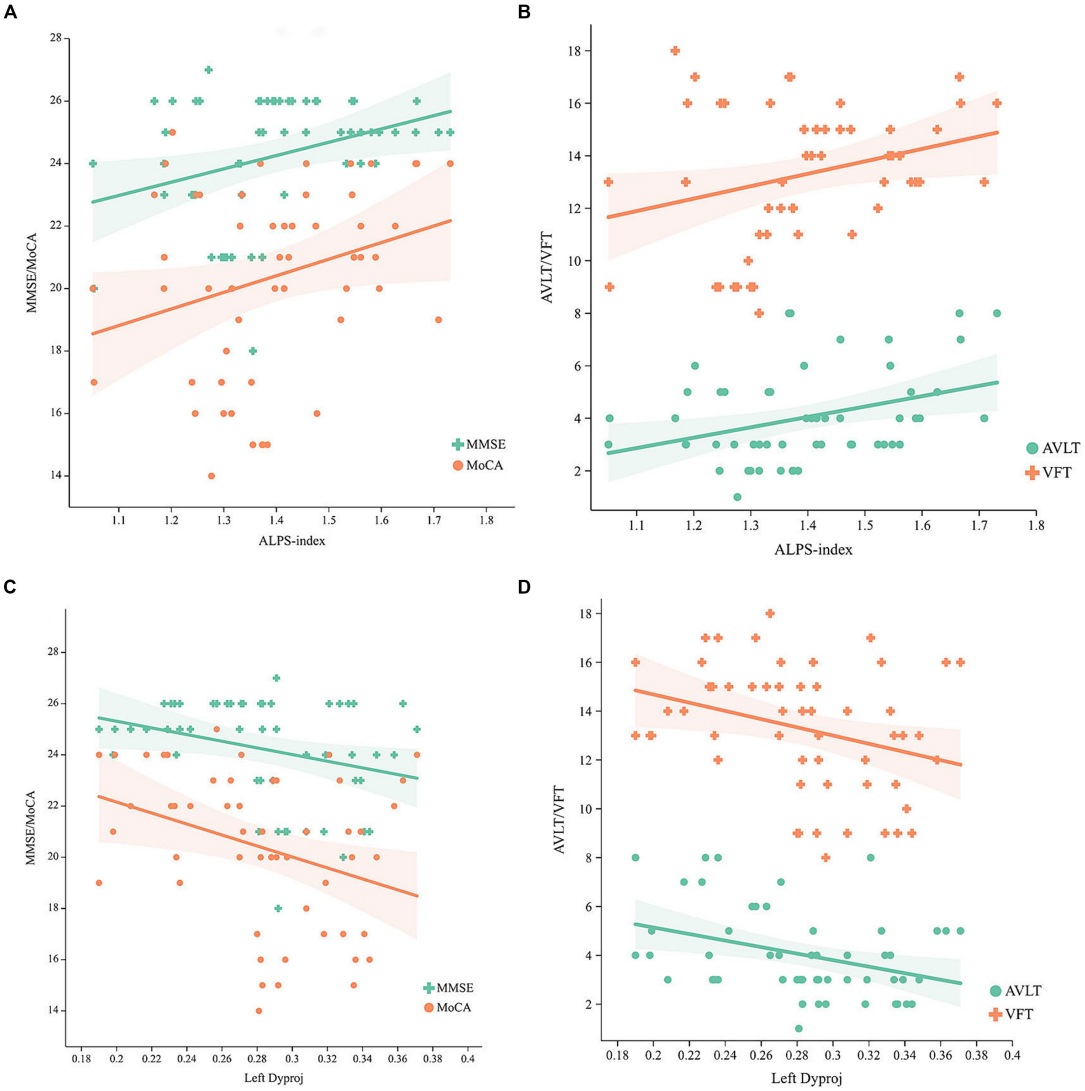
Correlation between the ALPS-index, left Dyproj and cognitive function in MCI. **(A)** Correlation between the ALPS-index and MMSE, MoCA scores. **(B)** Correlation between the ALPS-index and AVLT, VFT scores. **(C)** Correlation between the left Dyproj and MMSE, MoCA scores. **(D)** Correlation between the left Dyproj and AVLT, VFT scores. ALPS, diffusion tensor imaging along the perivascular space; Dyproj, diffusivity along the y-axis in projection fiber area; Diffusivity was presented as apparent diffusion coefficient values (× 10^−3^ mm^2^/s); MMSE, mini mental state examinations; MoCA, Montreal cognitive assessment; AVLT, auditory verbal learning test; VFT, verbal fluency test.

Adjusting for age, gender, and TIV, the ALPS-index was positively related to general cognition (MoCA, *r* = 0.274, *p* = 0.045; MMSE, r = 0.293, *p* = 0.031) (Figure 2A), verbal fluency (*r* = 0.273, *p* = 0.046) and memory (*r* = 0.327, *p* = 0.016) (Figure 2B), but the left Dyproj was negatively related to general cognition (MoCA, *r* = −0.362, *p* = 0.007; MMSE, *r* = −0.317, *p* = 0.02) (Figure 2C), memory (*r* = −0.369, *p* = 0.006) and verbal fluency (*r* = −0.344, *p* = 0.011) (Figure 2D). The results of the correlation analysis indicate a higher ALPS-index is associated with higher cognitive function in MCI, and a higher left Dyproj is associated with lower cognitive function. It is proposed that glymphatic function plays a protective role in cognition.

### Relationship among glymphatic activity, CR and cognitive function

**Figure 3:**
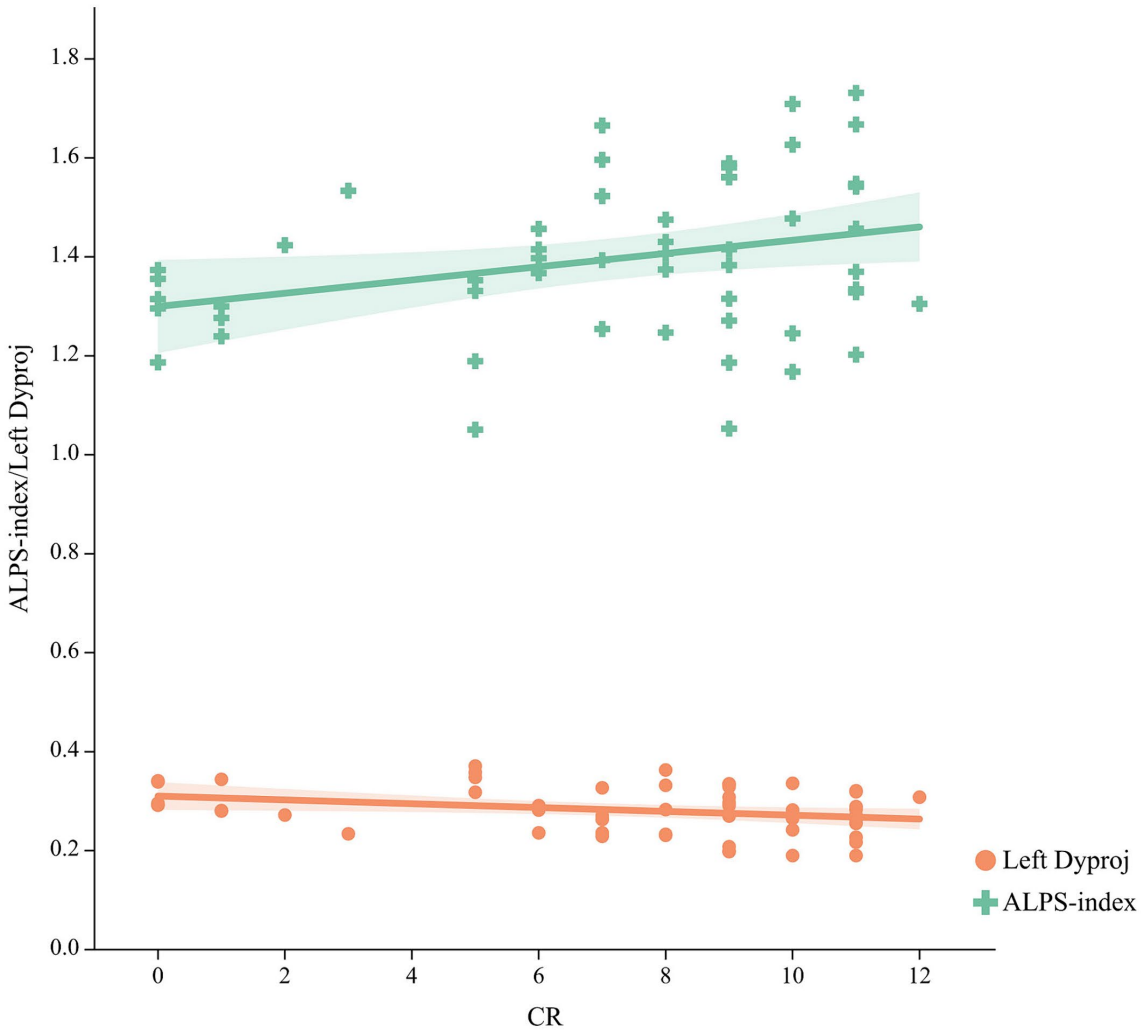
Correlation between the CR and ALPS-index, left Dyproj in the MCI group. CR, cognitive reserve; ALPS, diffusion tensor imaging along the perivascular space; Dyproj, diffusivity along the y-axis in projection fiber area; Diffusivity was measured with apparent diffusion coefficient values (× 10^−3^ mm^2^/s).

**Figure 4:**
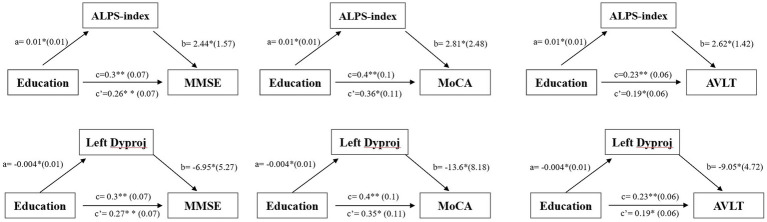
Simple mediation results of the effects of left diffusivity along the y-axis in projection fiber area/ALPS-index on the relationship between education and cognitive function in the MCI group; a, b, c, and c’ are path coefficients representing unstandardized regression weights and standard errors (in parentheses). The c path coefficient represents the total effect. The c’ path coefficient refers to the direct effect. All analyzed a, b, c and c’ paths were significant, *p < 0.05, **p < 0.001. ALPS, diffusion tensor imaging along the perivascular space; Dyproj, diffusivity along the y-axis in projection fiber area; CR, cognitive reserve; MMSE, mini mental state examinations; MoCA, Montreal cognitive assessment.

As shown in Figure 3, a significant positive relationship was found between the ALPS-index and CR (*r* = 0.292, *p* = 0.032), while a negative correlation was found between the left Dyproj and CR (*r* = −0.317, *p* = 0.02) in MCI. These findings indicate that there is an association between CR and glymphatic activity in MCI. To test this possibility, mediation analyses were conducted with cognitive score as the dependent variable, years of education (CR) as the independent variable, and glymphatic activity as the mediation variable. According to mediation analysis, the left Dyproj and ALPS-index partially mediated the effect of the relationship between education and cognitive performance. As described in Figure 4, an increase in ALPS-index was related to an increase in the cognitive score (path b). Moreover, changes in education affected glymphatic activity (path a). The mediation effect was assessed by path a × b = c- c’ and the mediation effect size was assessed by a × b / c. A positive relationship was found between the education and cognitive function, ALPS-index significantly explained 13.3, 10, and 17.4% of the overall effect of the link between the CR and MMSE, MoCA and AVLT score, respectively. An increased left Dyproj was related to a decreased cognitive score, and left Dyproj significantly explained 10, 12.5, and 17.4% of the overall effect of the link between CR and MMSE, MoCA and AVLT, respectively. The results further confirm the protective effect of CR on cognitive function, and suggest that this effect is partially achieved through the preservation of glymphatic activity.

## Discussion

In this study, we assessed the glymphatic activity in MCI and NCs by using DTI-ALPS and explored the relationship among glymphatic activity, CR, and cognitive performance. We found that MCI exhibited decreased glymphatic activity compared to NCs and CR has a protective effect against cognitive decline in MCI, and this effect may be partially mediated by changes in glymphatic activity.

Consistent with previous results (Steward et al., 2021), we found that lower ALPS-index was related to lower cognitive test scores in MCI patients. Recent evidences have suggested that compared with the NCs group, the volume and density of virchow-robin space (VRS) in MCI patients were significantly increased (Niazi et al., 2018) and increased VRS was related to the changes in CSF flow and decline of the waste clearance system (Sepehrband et al., 2021). A possible explanation for this might be that the enlargement of VRS could lead to expansion of impaired glymphatic flow, which reduce the removal of metabolic wastes and increase the concentration of Aβ and Tau protein (Rasmussen et al., 2018). As mentioned in the positron emission tomography (PET) study, ALPS-index was negatively related to the deposition of amyloid protein and microtubule-associated protein tau, and was positively correlated with cognitive scores in several AD-related brain regions (Hsu et al., 2023). Besides, lower ALPS-index was correlated with lower deposition of Aβ42 in CSF and fluorodeoxyglucose-18 uptake, and worse multiple cognitive impairment in MCI (Kamagata et al., 2022).

We also found that bilateral Dyproj values in MCI group were increased compared to the NCs group, and were negatively correlated with cognitive test scores. This finding was also reported by Taoka et al. (2017) which may be associated with the degeneration of WM in the projection fibers as a result of MCI (Fellgiebel and Yakushev, 2011). A study on DTI mapping also showed increased axial and radial diffusivity of projection fiber in patients with AD, which may be related to a reduced tissue density of the fiber (Huang et al., 2012). Similar results were found in a previous study, they found that Dzassoc and Dyproj were significantly increased in AD patients when compared with the NCs, revealing increased water diffusion perpendicular to association and projection fibers, respectively (Kamagata et al., 2022). The absence of significant changes in Dxxassoc and Dxxproj values reflect that the increase in water diffusion perpendicular to the WM tracts is offset by a decrease in water diffusion along the perivascular spaces (PVS). These findings demonstrated that the degeneration of these fibers in AD and glymphatic activity in MCI stage of pre-AD may be impaired, and the ALPS-index may reflect the glymphatic function which is influenced by the extracellular microenvironment.

As expected, we further found that a significant positive relationship between the ALPS-index and CR, while a negative relationship was observed between the left Dyproj and CR in MCI group, and glymphatic activity mediated the effect of the relationship between CR and cognition. This finding broadly supports that the CR was not directly related to cognition, but rather that its influence on cognitive function was partially mediated by glymphatic activity. It also further confirms that CR can not only modulate brain structure and functional network in MCI, but also has an effect on glymphatic activity. In accordance with the present results, previous studies demonstrated that the higher CR may moderate the negative impact of cerebral small vessel disease on cognitive function (Durrani et al., 2021) and the correlation between cerebral blood flow and category fluency is influenced by CR in MCI (Brenner et al., 2023). Early studies also indicated that the protective role in education against cognitive decline in MCI is mediated at least partially by changes on cerebral blood flow in right hippocampus (Zhu et al., 2023). This finding was also reported in experiments on young-onset AD to show that the relationship between the ALPS-index and cognitive function was significantly mediated by gray matter reserve (Chang et al., 2023). Similarly, Hisao et al. found that a higher ALPS-index suggested a stronger cortical reserve in areas that align with the default mode network, lower ALPS-index and cortical atrophy in specific regions were related to decreased mental manipulation and short-term memory among older individuals (Hsiao et al., 2023). It is somewhat surprising that we only found MCI groups had significantly lower right ALPS-index, and a negative correlation between the left Dyproj and CR, there is no difference on left ALPS-index between two groups and no correlation between right Dyproj and CR. It seems possible that these results are due to the relatively small sample size may not accurately reflect the correlation. Another possible explanation is that the ROI was manually placed, which could introduce subjectivity into our measurements and account for the observed differences.

These findings may be somewhat limited by the relatively small number of subjects, the relationships between CR, ALPS-index, and cognitive function may be different in more diverse individuals. Another potential limitation is the CR only used education as a proxy, but CR was also associated with other factors such as occupational attainment and cognitive activity. Therefore, future studies should take more CR proxies into account for a more accurate analysis. Finally, the ALPS-index was calculated from the whole brain average, which did not reveal regional glymphatic impairment, further studies evaluating regional glymphatic dysfunction in MCI-related brain regions are needed.

## Conclusion

The results of this research support the idea that glymphatic activity in MCI patients was lower than NCs and suggest a role for CR in moderating the associations between glymphatic activity and cognition. Further modeling work will need to be conducted to expand the sample size and assess the regional glymphatic activity using more CR proxies.

## Data availability statement

The original contributions presented in the study are included in the article/supplementary material, further inquiries can be directed to the corresponding author.

## Ethics statement

The studies involving humans were approved by the Ethics Committee of Lanzhou University Second Hospital. The studies were conducted in accordance with the local legislation and institutional requirements. The participants provided their written informed consent to participate in this study.

## Author contributions

LZ: Conceptualization, Formal analysis, Writing – original draft. WY: Data curation, Investigation, Writing – review & editing. YL: Data curation, Investigation, Writing – review & editing. YZ: Investigation, Methodology, Writing – review & editing. XG: Data curation, Formal analysis, Writing – review & editing. KA: Software, Writing – review & editing. GL: Supervision, Writing – review & editing. JZ: Funding acquisition, Supervision, Validation, Writing – review & editing.

## Footnotes

^1^ https://zenodo.org/record/3937147

^2^ https://rii.uthscsa.edu/mango/mango.html
